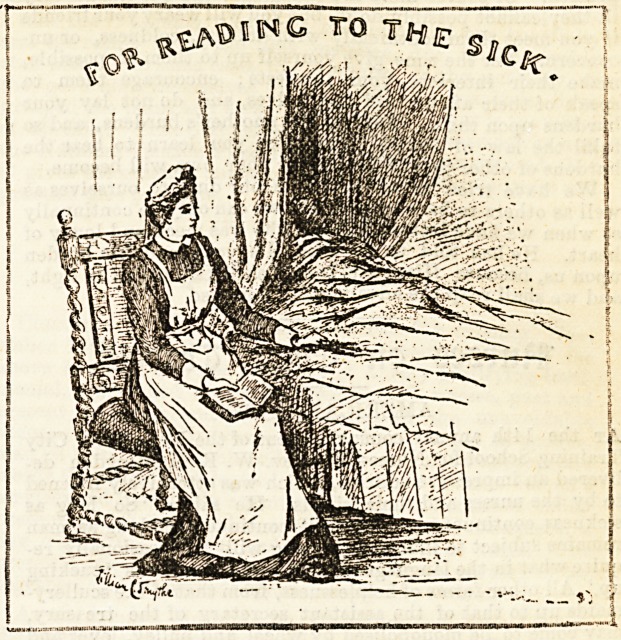# Extra Supplement—The Nursing Mirror

**Published:** 1890-12-06

**Authors:** 


Being the Extra Nursing Supplement of "The Hospital" Newspaper.

All Contributions for this Supplement should be addressed to the Editor, The Hospital, 140, Strand, London, W.C., and should have the word "Nursing" plainly written in left-hand top corner of the envelope.

### En Passant.

SHORT ITEMS.—The Sisters of Charity of Lanark Hospital wrote to us for a free copy of the "Annual," which we gladly sent them; their note concluded: "The funds of the hospital are very low." We recommended the consideration of that phrase to our Catholic readers.—Miss Louisa Twining writes in the *Nineteenth Century* on "Women as Public Servants."—Miss Arabella Kenealy writes in the *National Review* on "The Talent of Motherhood."—A correspondence has been going on in the *Wilts County Mirror* in favour of the extension of Miss Broadwood's system of Cottage Nursing.—Mrs. Torbett has been appointed matron of the new crêche at Neston, Cheshire.—Miss Harriet M. Kimball has earned a prize of £20 for the best hymn for use in the hospitals of the United States.—A hot correspondence is going on in the medical journals on the subject of the "Midwives Registration Bill."

NURSING HEROINES.—Last week's *Queen* contains a portrait and life of Miss Amy Potter, who is going out as matron-nurse to the hospital at Larnaca, Cyprus. Miss Potter is a granddaughter of the Rev. Dr. Potter, of York; she trained at Marylebone Infirmary, where she passed first at the examinations. In 1888 Miss Potter went to Southampton as head nurse to the female wards, and here she won the golden opinions of Dr. Percy Lewis, who speaks of her as "a perfect nurse."—This month's *Sunday at Home* contains further particulars of Miss Kate Marsden's career, telling what difficulties she went through at Bucharest, and how she won the order of the Red Cross Society of Russia. Miss Marsden has lately seen the Empress of Russia and taken farewell of her, ere starting for Siberia. The thoughts and prayers of many nurses will be with Miss Marsden during the next few months.

RARE COURAGE OF A NURSE.—The career of the Homerton Fever Hospital has been marked by one scandal after another; only on February 8th of this year we called the attention of the Local Government Board to the abominable mismanagement, and now a special committee has been called to consider the statements of a late patient, who complains of the food and treatment he received at Homerton. Possibly once more oblivion might have swallowed up the complaint; but, with rare courage, a nurse has come forward, and, in a long letter to the committee, has given evidence in support of the patient's complaints. The courage of nurses is proverbial, but still it needs a very brave woman to come forward in such an unheroic cause as that Nurse D. Halkin has taken up, for no laurels or thanks will be hers, and her life will be a trying one for many a day to come. The evidence of Nurse Halkin is briefly this: The food was extremely bad at the time the particular patient was in, and complaints to Dr. Collie brought no redress; the milk was often sour, the beef-tea made of tainted meat, and therefore diarrhœa prevailed; stimulants were provided in dirty, uncorked bottles; patients suffering from diphtheria did not have their throats examined; albuminuria following scarlet fever was not detected by the medical officer; the bedding is not properly disinfected; the nurses are, many of them, very young and inexperienced, and allowed to manage their wards as they like; the officers of the institution do not take proper care to avoid spreading infection; the clothing for the patients is insufficient. Here, then, is a definite and detailed indictment which the local board surely dare not ignore; an absolutely efficient inquiry must immediately be organised, and the past scandals must be reinvestigated, so that the root of the evil be reached. Be it noted that the matron resigned last January during the late scandal, and that the present matron has only been a few months at her post. We think the thanks of all nurses, and of all the people of England, are due to Nurse Halkin for her courageous statement. Several complaints have reached us from Homerton Fever Hospital at different times, but the writers never had the courage of their opinions, and we were therefore powerless to give proper help in the matter.

BELFAST NURSES' HOME.—On November 28th the annual meeting of this nurses' home and training-school was held, there being a good attendance. Professor Whitla, M.D., read the report, which stated that the nurses were trained as well as in the great London schools, and had lectures and classes. They had lately adopted a distinctive uniform. The finances were low, because the nurses had suffered severely during the past year from influenza; the bank account is overdrawn £1 6s. 4d. The nurses are trained in the Belfast Royal Hospital, and their work is much appreciated. Mr. J. J. Murphy, the Rev. Dr. Hanna, and the Rev. John Spencer, all praised the nurses and the management of the Home.

NURSES' FOOD.—In response to the request of two correspondents we reproduce the summary of articles used at the chief hospitals for the nurses' meals, from the paper lately read before the Hospitals Association. This ought to be useful to matrons who are seeking in what direction to add variety to the diet of the nursing home.

Day Nurses' Menu.

*For Breakfast.*—Tea, coffee, cocoatina, bread and butter, bacon and eggs (in one case new-laid eggs are specially imported from Lincolnshire), ham or tongue, haddocks, sardines, kippers, potted lobster, marmalade, potted meat, pickled pork, sausage-rolls, cold bacon, and broiled bacon.

*Lunch.*—Beer, coffee or milk, cheese, pudding, bread and butter, treacle, odds and ends.

*Dinner.*—Soup, meat, hot dishes (two courses), stews, beefsteak pie, fish, haricots, rabbits, geese, chops, made dishes, cold joints (in summer with salad), occasionally poultry and game; vegetables (two kinds), puddings, stewed fruits, beer, porter, milk, and cheese.

*Tea.*—Bread and butter, cake, jam, marmalade, treacle, sometimes salad.

*Supper.*—Soup, meat (cold or hot), puddings, milk pudding, salad, bread and cheese, hash with vegetables, sausage or fish pie, mince, fried bread and bacon, scones and cheese, porridge, made-up dishes, curries, pickles with cold meat, coffee, beer, porter, milk, bread.

Night Nurses' Menu.

*Supper or Breakfast.*—Soup, meat, eggs, fried bacon, mince and mashed potatoes, stew, sardines, cold bacon, broiled bacon, fish, puddings, marmalade, bread and butter, tea, coffee, milk, and beer.

*Dinner.*—Hot joints, cold joints, or made dishes, fish, steaks, chops, cold joints (in summer with salad), vegetables, puddings or tarts, bread and butter, cheese, beer, porter, coffee, or milk.

*Tea or Luncheon.*—Bread and butter, cheese, cake, jam, treacle, occasionally tea, ale, milk.

*Night Meals taken in Ward Kitchens.*—Tea, coffee, or cocoa, fish, eggs, meat, bacon, sausages, kippered herrings, German sausage, corned beef, boiled bacon, meat pie, curries, cutlets, sardines, bread and butter, vegetables, puddings, cold meat, jam.

### Lectures on Surgical Ward Work and Nursing.

By Alexander Miles, M.B. (Edin.), C.M., F.R.C.S.E.

---

### Lecture VI.—DRESSINGS.

There are still one or two substances with which you should be familiar before beginning to work in surgical wards.

Oiled Silk or Protective consists of thin sheets of oiled silk coated on both sides with copal varnish, which renders the silk impervious to lotion. Over this a layer of carbolized dextrine is painted. This material is unfortunately not antiseptic, because the carbolic soon volatilises from the dextrine. It is not used nearly so much nowadays as it used to be. When used it is placed over the wound to prevent adhesion of the other dressings, and to facilitate the escape of discharge, which soaks out all round the edges, and is thus more evenly distributed in the wool. When a drainage tube is being used, a hole is cut in the protective and the tube drawn through it. The tube is then transfixed with a safety pin to prevent it slipping out of reach. The protective also prevents the pin irritating the wound. In dressing large *healing* ulcers of all kinds, but especially those resulting from burns, it is of great importance that the margins should be very gently dealt with. It is here that the healing process is going on, and if you look carefully you will see the thin delicate blue line of young epithelium all round the sore. It is evident that if you apply any dressing which will stick into the wound this delicate epithelium will be removed at each dressing, and the healing process consequently retarded. To prevent this, then, you should cover this growing epithelial margin with thin strips of protective carefully purified.

Gutta-percha Tissue.—As its name implies, this is a very thin sheet of gutta-percha. It is used to put outside deep dressings or fomentations when you desire that these should remain moist, as it prevents evaporation of the fluid in the dressing. Non-antiseptic, it requires purification before being used, but you must be careful not to dip it into *hot* lotion, which will dissolve it. Excellent finger-stalls can be made with gutta-percha tissue one or two layers thick. The edges are easily fixed by a drop of chloroform, or better still, by a lighted match. I have found it useful also for making small ice bags to apply to a hernia-cerebri, and for other purposes.

Mackintosh or Pink Jaconette is thin cotton cloth with a layer of india-rubber waterproofing over it. Like gutta-percha tissue, it is used to prevent moist dressings becoming dry. Its chief use, however, is to protect the patient's clothing and the bed from being soiled during an operation or at dressing. For this purpose it is cut into sheets, about a yard square, the edges being left unhemmed. You must never fold up a macintosh while damp, as the adjacent surfaces adhere and ruin it. Carefully avoid sticking pins into macintosh, as the holes made allow lotion to run through, and permit of evaporation from fomentations, &c.

Gauze.—(1) *Antiseptic gauze.* This is a rough unbleached muslin, which has been rendered antiseptic by being charged with a mixture of carbolic acid, resin, and paraffine. The paraffine prevents the gauze adhering, while the resin fixes the volatile carbolic acid, and so prevents, to some extent, the evaporation of it, so long as the gauze remains dry, and below the temperature of the body. It used to be employed as a deep dressing, but is now almost entirely used for making bandages. (2) *Plain gauze* or *surgical gauze* is a loose cotton cloth, made absorbent by having its oily matter removed by boiling in soda. It is soft, open, and porous, and so, for its bulk, absorbs a large amount of discharge. It is cut into strips, and folded so as to make pads about four inches square, and six or eight layers thick. It is rendered antiseptic by being kept in 1 in 20 carbolic for about a fortnight before being used. Before being applied to the wound as a deep dressing the pad should be wrung out of corrosive, or some other less irritating antiseptic, as the carbolic is apt to irritate the skin. These pads will be found very useful as they can take the place of protective, or be used as a deep dressing, or even as sponges.

Bandages and Slings will be treated of later. Suffice it now to say that there are certain bandage materials which are antiseptic, for example, carbolized gauze, double cyanide of mercury and zinc gauze, and domette which has been impregnated with sal-alembroth; and others which are non-antiseptic, such as plain cotton and ordinary domette, and these should be used according as an antiseptic is indicated or not.

Lotion Basins, Trays, &c.—Before going further it may be convenient that I should indicate to you the various tins, basins, and other appliances usually found in a surgical word, and the uses to which these are put. (1) *Lotion basins.* These may be made of plain tin, or of tin which has been

(Fig. 1.)

covered with a coating of enamel. The former are to be used only for carbolic or boracic lotions, as corrosive destroys them. The enamelled tins are specially made for corrosive lotion, but of course are equally suitable for the others. The basins are of various sizes, and the size you select will depend on the quantity of lotion you expect to be used. Never fill the basin more than half full, and never take more lotion than is necessary. (2) *Solution tins* differ from lotion basins in having perpendicular sides, and in being provided with a handle. They are made of plain tin, and are used to steep sponges, towels, &c., in carbolic lotion. They are often used also to hold pus, serous fluid, or other discharges, and for various other purposes. (3) The *Kidney-shaped basin* (Fig. 1) is described by its name. It is a shallow tin or enamelled vessel, used to catch up discharges as they escape, *e.g.*, pus from a large abscess. On account of its shape it can be accurately applied to the surface of almost any part of the body, so preventing any soiling of the patient's clothes or sheets Always remember that before allowing it to go near

(Fig. 2.)

any wound whatever, it should be thoroughly purified with carbolic (1 in 20) lest it carry germs on to the wound. (4) *Bleeding cups* (Fig. 2). These small saucer-shaped dishes, although not now used for their original purpose, are still very useful, and should be found on every ward table. They are made of tin, and are usually graduated, so that the quantity of discharge caught in them may be measured. They are often used in place of the kidney-shaped basin, the same precaution being taken with regard to purifying their edges. They are found useful receptacles for the parts removed at operations, *e.g.*, excised parts of joints, tumours, and so on. (5) *Leg tray.* This is a long, shallow, oval tray, used in dressing wounds of the leg. At the upper end is a broad flange hollowed out to permit of the thigh resting on it, while the lower part of the leg lies over the tray, into which all discharge and lotion escapes. (5) *Dirty-dressing tray.* This is a large shallow tray, placed under the bed or operating-table to receive the dressing removed from the patient. When the dressing is finished, it should *at once* be removed from the ward, and emptied.

(*To be continued.*)

### Nursing Medals and Certificates.

No. I.—THE ROYAL RED CROSS.

THIS order for women was instituted by Queen Victoria in 1863, for the purpose of rewarding the services of those devoted to the sick and wounded in war. It is not confined to British subjects, and has been conferred on foreigners; it is worn on the left shoulder. The receiver of this order has to sign an undertaking to give it up should she in any way disgrace herself. The following are the names of those on whom Her Majesty has conferred this order:—

PRINCESSES.—H.R.H. the Princess of Wales; H.R.H. the Empress Dowager Victoria of Germany (Princess Royal of Great Britain and Ireland); H.R.H. the Princess Christian of Schleswig-Holstein (Princess Helena of Great Britain and Ireland); H.R.H. the Princess Beatrice; H.R.H. the Duchess of Connaught and Strathearn; H.R H. the Duchess of Teck; H.R.H. the Princess Louise, Marchioness of Lorne; H.R.H. the Duchess of Albany; H.R.H. the Princess Frederica of Hanover, Baroness Von Pawel Rammingen.

LADIES.—The Lady Wantage; Miss Nightingale; Miss Margaret Maistre, for services at Brunkers Spruit; Mrs. M. J. W. Lumley, Durban and Pietermaritzburg; Mrs. Gildea, Pretoria.

NURSING SISTERS OR OTHER PERSONS ENGAGED IN NURSING DUTIES.—Mrs. J. C. Deeble for services in Zululand; Miss A. E. Caulfeild (Lady Superintendent of Nurses, Herbert Hospital, Woolwich), Egypt and Transvaal; Miss H. Stewart (Superintendent of Nurses), Egypt, Hospital Ship "Carthage"; Mrs. M. A. Fellowes, Egypt, Hospital Ship "Carthage"; Miss J. A. Gray, Zululand and Egypt, s.s. "Lusitania"; Miss H. Campbell Norman (Lady Superintendent of Nurses, Royal Victoria Hospital, Netley), Egypt; Miss J. Jerrard, Transvaal and Egypt; Miss E. Cannell, Cyprus and Egypt; Miss M. Jones, Egypt; Miss B. Story, Egypt; Miss S. Airy, Cyprus and Egypt; Miss E. Wheldon, Netley; Miss J. M. Gray, Transvaal; Miss Langlands, Transvaal and Zululand; Sister Louise, Transvaal and Zululand; Sister Maria Celestina, Egypt; Sister Maria Pia, Egypt; Sister Maria Camilla, Egypt; Sister Maria Carmela, Egypt; Sister Maria Ludovica, Egypt; Sister Maria Benedetta, Egypt; Sister Camilla Orpheline, Egypt; Miss M. Selby, Zululand and Egypt; Miss J. King, Egypt; Miss K. Forrest, Egypt; Miss M. Thomas, Egypt; Miss A. Crisp, Zululand and Egypt; Miss A. Yardley, Egypt; Miss A. K. Holland, Egypt; Mrs. Janet King, Zululand; Miss Hornor, Zululand; Miss M. C. Jerrard, Egypt and Soudan; Miss H. King, Egypt and Soudan; Miss S. Ireland, Egypt and Soudan; Miss J. M. C. Barker, Egypt; Miss S. F. Hart, Egypt; Miss M. C. F. K. Cole, Egypt, Hospital Ship "Ganges"; Miss R. M. Burleigh, Egypt, Hospital Ship "Ganges"; Miss L. Parsons, Egypt; Miss A. Hind, Egypt; Miss C. L. Byam, Soudan; Miss R. Williams, Egypt and Soudan; Miss Louisa Jane Mackay, Transvaal and Egypt; Miss Christina Fergusson, Egypt; Miss Augusta Bailey Holland, Netley, Chatham, and Portsmouth; Miss Edith King (Stafford House Committee), Zululand and Operations against Sekukuni; Miss Emma Durham (Stafford House Committee), Zululand and Operations against Sekukuni, and Egypt.

THE DUTY OF CHEERFULNESS.

WHAT a very warm and comfortable feeling there is about the heart when we have done our duty with cheerfulness! Everybody has his work cut out for him. Sometimes an idle body is apt to let the work get done as it can, or at others to do it with a grudging, grumbling spirit.

What is it that makes everything seem easy and no trouble to us? Cheerfulness. For instance, we will look at the different way people take the same misfortune. Two men have been sadly injured by an accident, both are recovering. The doctor tells them they must move their injured limbs as much as possible, or they will become stiff and useless. One, with many sighs and moans, tries to walk; the pain is so intense, he says, that he cannot persevere, and he gives up in despair, with endless lamentations over a misfortune which will prevent his earning his bread in the future, or having any more pleasure in life. Foolish fellow! he only wants what men call pluck, that is, a little cheerfulness. His neighbour, however, who is a warmhearted, comfortable soul, takes the advice in the right spirit, he moves his leg a little one minute, then stops, then goes on again, and never gives in till he has by little and little overcome the stiffness, and he becomes as sound and hearty as before. It is no use sitting down and making our moan when we should be up and doing. Cheer up, my dismal friend, and think of all the comforts that are left us even when at the worst. There is always a crook in my lot, says the downhearted man. Every cloud has its silver lining, says the cheerful one. Happily, there are few of us who cannot count a good many mercies if we only take the trouble to look for them. Above all there is the Friend who sticketh closer than a brother, who never fails us at a pinch, but does for us all, more than we really need, though not quite perhaps in the way we like or expect. He will help us to gain true cheerfulness, which springs from a heart satisfied and at rest in God. I do not mean a manner which can be put on for a time in order to hide the fierce struggles of a murmuring spirit, which restlessness or a fretful tone will betray, but that true cheerfulness which makes us forget ourselves and be contented with what our Heavenly Father gives us. When once we have attained to that we shall have time to look about us and consider others, and try to make them happy too. For there is a great deal of selfishness in low spirits. It is I—I—I all through. I wish, I want, I covet. Ask God for a loving spirit that may make you put your neighbour's comfort before your own. Among your own family you may in time become a bright presence, cheering and healing and strengthening them.

When you are feeling weary and languid you can rouse yourself just to say a word of thanks or give a smile of kindness. Let your greeting be the same to all people, and not pleasant to a favoured few and gloomy to the rest of the world. We all know what a hard task it is to smile when we are disinclined, or to talk, or even to see anyone when depressed. Of course there are times when people are so very ill they cannot possibly do it, but you will weary your friends if you meet them continually with dulness, coldness, or unconcern. For the time give yourself up to them if possible, make their interests your interests; encourage them to speak of their affairs and themselves, and do not lay your burdens upon them. Bear ye one another's burdens, and so fulfil the law of Christ. The more you learn to bear the burdens of other people the lighter your own will become.

We have tried to see that it is our duty to ourselves as well as others to be cheerful, but we can only be continually so when we have learnt of Him who was meek and lowly of heart. He has told us to take His yoke and His burden upon us, because His yoke is easy and His burden is light, and we shall reap the advantage of doing so.

### Nurses on Their Travels.

### AMERICAN NOTES.

At the 14th annual commencement of the New York City Training School for Nurses, the Rev. W. R. Huntingdon delivered an impressive address, which was attentively listened to by the nurses and their friends. He said: "So long as sickness continues, nursing will continue. So long as man remains subject to infirmity, so long will he occasionally require what in the language of childhood is known as 'tucking up.' All other forms of helplessness, from that of the scullery-maids up to that of the assistant secretary of the treasury, may come to be monopolised by wheel and pulley, lever and cam, boiler and dynamo, but the service of the sick will continue human. Nurses will always be in demand. But although nursing is thus, in the strict sense of the word, a handicraft, we are not to forget that it is something more and better than this: it is an art. This is what we have in mind when we talk about trained nurses' in distinction from un-trained ones. The fact that some women are, as we say, 'born nurses' no more disproves the assertion that nursing is an art than the fact that some men who were never taught to ride do nevertheless acquit themselves in the saddle extremely well disproves there being such an art as the art of horsemanship."

Some time since we gave particulars of the Directory for Nurses at 249, Vermont Street, Buffalo, New York; and now we get the following letter from a nurse who trained in London, but is now working out there:—

"Miss Sarah Sheldon, who has charge of the Directory for Nurses here at the Buffalo Medical College, has had letters from English nurses asking about the private nursing, and as I am the only English nurse out of about one hundred nurses who have their names on the directory, I thought I would write a letter to The Hospital. I have been here two years; this is a lively city, and I like private nursing very much. I have always found the people I have nursed for very kind, and my patients very grateful for what I have done for them. The doctors are very considerate for their nurses. We are allowed one hour every day for out-door exercise, and from five to six hours' continuous sleep, more if possible, and get from ten to fifteen dollars every week. If any nurse would like to try private nursing in Buffalo I should be glad to give any information I can."

The nurses of the Bellevue Hospital have formed themselves into an Alumnæ Association, and hold monthly meetings. A paper is read, and a discussion follows. The last paper, read on October 30th, was by Miss Merritt, on "Loyalty," and its object was to discover the reason of the present disloyalty and discontent amongst nurses. Miss Merritt believed that implicit obedience should be taught as part of a nurse's training, and this would, perhaps, crush the grumbling, carping spirit of the age.

Granting Canada to be a part of America according to the map, we would mention here that Miss Stone, of Montreal, is again giving an excellent course of nursing lectures, which are well attended, and which are reported in the *Montreal Gazette*. Miss Stone lays great stress on the necessity for keeping the sick-room bright and cheerful.

The Philadelphia Directory for Nurses has 800 nurses on its list; last year there were 1,006 calls for nurses in the city, and 320 out of the city. The finances are satisfactory. Dr. Weir Mitchell gets all his nurses through this directory. The nurses take their own earnings and pay a percentage to the office.

### The Trained Nurse in the Nursery.

### II.—A Day's Routine in Private Nursery. (Two Nurses two Children).

At a quarter before seven in the morning your under-nurse comes in to see if anything is required, or if you want a fire to dress the children by (after she has done her day nursery grate).

You then get up, and by the time your maid has finished the day nursery and set the breakfast table, you will be ready when she brings the bath water to bath and dress the children, taking the elder first. You bath him, dry him, and put on some of his clothes, leaving the maid to finish, whilst you do the baby, who will take longer.

When the under-nurse has finished the little boy, she turns up the beds, empties the slops, until you are ready. Then you take the children into the day nursery. Whilst your maid fetches up your breakfast, you give baby her bottle and make the boy's bread and milk; he can then take his breakfast while you are having yours. Your little boy fed and comfortable, your baby asleep, after breakfast you make your bed, or help to do it, wash your bottles for the day, make the food for baby, put baby's night garments ready, and, if your children are fairly good, you will get a little quiet to yourself before it is time to take them out. At half past ten you give your boy a biscuit and some milk, your baby a fresh bottle, and dress yourself for walking. Your under-nurse in the meantime has washed up breakfast things, done night nursery, made her own bed, and is able to go with you to wheel the perambulator, etc. If your lady has not been up to see the children, take them to her on your way out. Ask her if she has any orders to give; ask for any stores you may require; tell her anything about the children you think will interest her. Then take your bairns for a good two hours' walk, bring them in fresh and bright about half-past twelve, in time to get them undressed for dinner at one o'clock. It will save you much trouble to feed your boy before getting your own dinner; he will then play about quite happily if fed and warm; or his mother may wish to have him at her luncheon, in that case your maid will take him down when she fetches your dinner.

At two p.m. put your little man to bed for a nap, and, if possible, your baby also. You will then have time to get ready their evening frocks, sashes, &c., before again taking them out.

At three p.m. take both children for a walk until half-past four, when you will bring them in to tea. After tea change your baby's frock, whilst the maid dresses the boy, and take them to the drawing-room at half-past five to their mother. Most mothers keep them for at least an hour, when the nurse can make the baby's food for night, do any repairs quietly; but the main sewing days are wet days.

At a quarter past six, fetch your baby, put her to bed before sending the under nurse for the boy; both children ought to be in bed by seven p.m.

You will often find then little motherly things to be done: a frock to be ironed out to last another day, a best shawl to wash, etc., but at eight p.m. you may fairly consider your day's work done.

Most nurses go to bed at ten o'clock, and before retiring, take the boy up, give him a drink and make him comfortable for the night. Feed your baby, and put the night's supply of food ready, with a night-light for the infant.

Your under nurse having cleared up the day nursery and supper things, you dismiss her with a kindly good night, and though you have nursed no great case, or done anything what the world would call heroic you have earned the Master's "well done," though your day has been spent in only caring for two babies. But I myself deny the "only," for even Christ Himself, in His last days upon earth, gave us the command to care for the little ones; and as God has created them the most helpless of all His creatures, they demand our tenderest care and love, although it entails a life of self-denial and patience. Sister Grace.

### Everybody's Opinion.

[*Correspondence on all subjects is invited, but we cannot in any way be responsible for the opinions expressed by our correspondents. No communications can be entertained if the name and address of the correspondent is not given, or unless one side of the paper only be written on.*]

### THE GENERAL BONUS FUND OF THE NATIONAL PENSION FUND FOR NURSES.

"One in Charge of Hospital-trained Private Nurses" writes: In an article headed "Pensions for Hospital Workers," signed "Agricola," in The Hospital, of November 22nd, 1890, the writer, speaking of the National Pension Fund for Nurses, says, "A Bonus Fund, the interest upon which will go to swell the pensions, has been started, and with a munificence calling for especial acknowledgment, donors have come forward with offerings amounting in all to £40,000. *This sum is a free gift to our hospital nurses, for to them the advantages of the Bonus Fund are limited.*" (The marks of emphasis are mine.) Surely this statement cannot be correct? I am certain that all the members of our institution who have joined the National Pension Fund did so under the conviction, confirmed by the officials of the Fund, that the "General Bonus Fund is to be used for the benefit of all nurses within the British Empire who may, from time to time, join the National Pension Fund" (see "Extract from the *Times*," July 8th, 1889, printed and distributed on behalf of the National Pension Fund). The expression "all nurses within the British Empire," is a very broad one, and entirely excludes the possibility of limiting the benefits of the General Bonus Fund to "hospital nurses." Perhaps "Agricola" means "hospital-trained nurses, which is a very different matter, although even here the limit is narrower than the official statement. Everyone will gladly admit that 'hospital' nurses richly deserve both pension and bonus, and all who know anything about their trained fellow-workers in the private and district nursing departments of the profession, are fully aware that these women equally merit the same benefits. I am a hearty well-wisher to The National Pension Fund, and to all who are in any way connected with it; whilst the fact of my not being a policy holder, should absolve me from selfish interest in the matter."

[All Nurses will participate.—Ed.]

Mr. Edward T. Clifford, manager of the Pension Fund, writes: In your issue of the 22nd inst., under the heading of "Passing Topics," appears an article signed "Agricola," on "Pensions for Hospital Workers." This article contains the following statement in reference to the National Pension Fund for Nurses: "This sum (£40,000) is a free gift to our hospital nurses, for to them the advantages of the bonus fund are limited, &c." I desire to take the earliest opportunity to point out that this statement is unauthorised and incorrect. A reference to the prospectus of the National Pension Fund will show that the Council of the Fund have not laid down such a policy of exclusion; on the contrary, their policy has been to make the benefits of the Fund available for all nurses. Let me ask your readers to turn to the annotation on page 126 of The Hospital (same issue) which deals with nurses and their pay. There is, Sir, as you point out, a disposition on the part of hospital authorities to help nurses with their contributions to the Fund. The authorities are recognising that the conditions of affiliation convey a distinct advantage to themselves, committing them to but little and relieving the institution of a grave responsibility at a comparatively trifling cost with the knowledge that permanence, satisfactory from all points of view, will result therefrom. If these conditions exist, how is it that nurses do not try to push their opportunities in this direction and secure affiliation by their respective hospitals as promptly as possible? The reasons are not far to seek, but amongst them are the lack of leadership and initiative. Self-constituted leaders there are whose chief function appears to be to work against the authorities instead of with them; these persons, however well-intentioned, are doing more harm than good. But the initiative should come from within. Take the case of the London Hospital. Here sixty women are provided for for life, as the governors have recognised the justice of their appeal. I take the "London," as it is the largest of our hospitals, and the result is, in the first instance, due to the nursing staff alone. In some cases the treasurer, or the secretary, or the governors themselves of the affiliating hospitals, have grasped the point and acted accordingly. What is the moral of all this? That God helps those who help themselves, and the National Pension Fund for Nurses is so constituted that it gives the greatest opportunities to those who recognise and practice that principle.

### Presentations.

Cornelia Hospital, Poole.—On Tuesday evening, November 18th, Mrs. H. Raven, the esteemed matron of the above hospital, was the recipient of a very gratifying testimonial, subscribed for by the medical staff, nurses, past and present patients, and other friends. In the unavoidable absence of Lady Wimborne, Dr. McNicoll acted as spokesman on behalf of the subscribers, and with a few appropriate words (in which he eulogised the character of Mrs. Raven's services and her kindly attention) handed to her the birthday present, which consisted of a handsome marble timepiece, a five o'clock tea service, and a pair of antique vases. The present patients, friends, and medical staff, all partook of tea together, and afterwards spent an enjoyable evening.

At the London Hospital, on December 2nd, Miss Lückes, the matron, was presented with a gold watch and bracelet, and a silver lamp, from ex-workers at the London Hospital. The presentation was made as a mark of sympathy and of appreciation of the aid given by Miss Lückes in forwarding the interests of the nurses and of nursing. Over one hundred nurses who owe their training to Miss Lückes, and many of whom are now matrons themselves, had subscribed to the testimonial. Such names as those of Miss Yeats, matron of Gloucester Infirmary; Miss Hetherington, of the Victoria Park Hospital; Miss Moir, of St. Pancras Infirmary; and Miss Paget, General Inspector of Nursing to the Queen's Jubilee Institute, show that those who have practical acquaintance of nursing thoroughly appreciate the work of Miss Lückes.

### Notes and Queries.

### Queries.

(18) *Massage.*—Where can I learn massage in Germany, and what will it cost? Will some nurse please answer?—*Brighton.*

(19) *Ear-drums.*—I should be glad to hear from anyone who has derived benefit from wearing silver ear-drums for deafness. Where can they be procured, and should an aurist be consulted before using them?—*Ears.*

### Answers.

*The Trolly Splint.*—We beg to inform "Lady Superintendent" that this splint can be bought from "Nurse," 71, Campbelle Road, Bow, London, E., price 5s., or to hospitals 2s. 6d.

*Nurse Gertrude.*—"The Management of Children" by A Mother, published by Churchill, is very good; we fancy the price is 7s. 6d. If it is solely nursing you want you might get "A Handbook for the Nursing of Sick Children," by Miss Wood. The first is the bigger volume and tells chiefly how to treat minor ailments and bring up infants.

*W. B. H.*—Thanks for the verses; we will use them shortly.

*C. H. L.*—Copies of the paper on "Nurses' Food, Work, and Recreation," can, we believe, be had from The Hospitals Association, Norfolk House, Norfolk Street, Strand, W.C.

*G. M. E.*—There is no hospital where you can learn monthly nursing without paying a premium, unless you join some large infirmary where there are lying-in wards; but in that case you would probably have to sign for two years. You can learn monthly nursing at the British Lying-in Hospital, Endell Street, in a month for a fee of £7.

*Nurse Winter.*—We should think there are plenty of nurses with a little money, who would be glad to buy your invention, since you have not the means to patent it yourself. Go and see the secretary, Midwives Institute and Trained Nurses Club, 12, Buckingham Street, Strand, any day between 2 and 7 p.m. She would perhaps allow you to put a notice on the notice board.

*Marguerite.*—Get the "Physiology" and "Anatomy" published by Macmillan, in their series of Science Primers, price 1s. each. There is a very popular little book, "The house I live in," published by Longmans, which treats these subjects in an interesting way, which might better please your scholars than the more concise primers.

*H. B. W. P.*—Artificial noses are made of different materials, but the best and cleanest are made of celluloid fixed to the face by means of glasses or only the spectacle frames, or other methods. Possibly Mr. F. Sillis, 2, George Street, Euston Road, N.W., could give you further information.

*"Burdett's Hospital Annual."—F. Horfield.*—This "annual" can be obtained at The Hospital Offices, 140, Strand, W.C., price 2s. 6d., or 2s. 9d. if sent by post.

### Domestic Medicine in the 18th Century.

It has been left for the 19th century to develope first the Sairey Gamp and then the efficient nursing system of the present day. An ailing world had been tyrannised over by incompetence for centuries and nauseated by the horrible drinks and decoctions lavishly poured down the throats of the afflicted, for the cure of the strange ailments which appear to have beset mankind in ancient times. Doubtless, even then, most women acted up to their light, which light, however, resembled the farthing rush candle of antiquity rather than our own gas jet, and we may be thankful that Charles Dickens arose to draw attention by his genial humour to the miserable state of things which still existed in his day.

We learn from Sir Walter Scott's novels that ladies were not wholly ignorant of the healing arts in the middle ages, and in the 17th and 18th centuries much was done by them with herbs and simples. Indeed, a lady's education was not considered finished till she had added practical cookery and domestic medicine to her other accomplishments.

We have before us a copy of "The Compleat House Wife, or Accomplished Gentlewoman's Companion" in its fourteenth edition, published in 1750. It is a thick octavo vol., bound in a stout leather cover, which has manfully withstood the wear and tear of 140 years, and is in itself an example of the survival of the fittest. It contains a collection of upwards of six hundred receipts in cookery, to which is added, and here we beg to draw the attention of the reader more particularly: "A collection of above Three Hundred Family receipts of Medicines, viz., Drinks, Syrups, Salves, Ointments, and various other Things of sovreign and approved Efficacy in most distempers, Pains, Aches, Wounds, Sores, &c., particularly Mrs. Stephens's Medicine for the Cure of the Stone and Gravel, and Dr. Mead's famous Receipt for the Cure of a Bite of a mad Dog; with several other excellent Receipts for the same, which have cured when the Persons were disordered and the salt Water failed; never before made public; fit either for private Families, or such public-spirited Gentlewomen as would be beneficent to their poor neighbours."

It will be amusing to cull a few prescriptive flowers from this garden of pharmacy.

*Place aux dames.* Let us first take notice of Mrs. Joanna Stephens's valuable prescription for stone and gravel. It was composed chiefly of egg-shells and snails, mixed with herbs, soap, and honey. These were divided into powders, decoction, and pills. The powder to be taken three times a-day in a "large tea-cup full of white wine, cider, or small punch, and half-a-pint of the decoction after every dose." In certain cases the person was to take five pills every hour (!) day and night, when awake. An excellent proviso, for it would require a strong mind to take, and a still stronger stomach to retain such a quantity of repulsive condiments; so probably the patient frequently feigned sleep to avoid it. The following is appended to the receipt:—

"N.B. Mrs. Stephens received five thousand pounds reward on her medicine having been tried and approved. March 17, 1739-40. See *London Gazette*, March 23, 1739-40."

*(To be continued.)*

### Where to Go.

Spohr's "Last Judgment" will be given at Marylebone Church on Thursdays, December 11th and 18th. Admission is by ticket only, which can be had free from the churchwarden.—During Advent special services are held at most of the churches, including services in the choir of Westminster Abbey every Sunday evening at seven.—The second series of lectures given by the Sunday Lecture Society begins tomorrow in St. George's Hall, Langham Place, at four p.m., when Sir James Crichton Browne, M.D., F.R.S., will lecture on "Brain Stress."—To-night (December 6th) Mr. A. N. Cumming lectures at the Working Men's College, Great Ormond Street.—Mr. Edward Solomon, the popular composer, gives a musical and dramatic soirée at the Princes' Hall, on the evening of Tuesday, December 16th. He will be assisted by a brilliant array of talent.—Until December 15th an exhibition of pictures will be open at 29, Queen-square, from three till six p.m. daily, under the auspices of the Art for Schools Association.—There will be a matinée of "Antony and Cleopatra" at the Princess's Theatre next Saturday, Mrs. Langtry as Cleopatra.—Madame Albani will sing in Mackenzie's "The Rose of Sharon," at the Royal Albert Hall, on Wednesday, December 10th, at eight.—A lecture and demonstration of special interest to nurses will be held at the Institute of Medical Electricity, 25, Fitzroy Square, W., on December 11th at three p.m. Mr. Newman Lawrence will lecture on "Medical Applications of Electricity," and give demonstrations on living subjects. Nurses will be admitted free by tickets, which can be obtained by sending a stamped addressed envelope to the Secretary at the institute.

### Lectures.

Guthrie Society, December 11th.—Clinical Cases, Mr. A. W. Harrison. M.R.C.S., L.R.C.P.; Paper by Mr. C. W. Glassington, M.R.C.S., L.D.S., on "Dentistry in Relation to Medicine and Surgery"; exhibition of surgical instruments, latest inventions, &c.

### Appointment.

Hospital for Sick Children, Great Ormond Street.—Miss Close, of the Convalescent Home, Kingston, was on Tuesday last appointed lady superintendent of this hospital, in place of Miss Philippa Hicks, who has resigned after two years of excellent work. We are sure Miss Close will find her new post a pleasant one, and we congratulate her on her success.

### Amusements and Relaxation.

**N.B.—THIRD QUARTERLY WORD COMPETITION**

**Commenced Oct. 4, 1890; ends Dec. 27, 1890.**

Three prizes of 15s., 10s., 5s., will be given for the largest number of words derived from the words set for dissection.

N.B.—Word dissections must be sent in WEEKLY not later than the first post on Thursday to the *Prize Editor*, 140, Strand, W.C., arranged alphabetically, with correct total affixed.

The word for dissection for this, the TENTH week of the quarter, being "ATLANTIC."

| Names. | Nov. 27th. | Totals. | Names. | Nov. 27th. | Totals. |
|---|---|---|---|---|---|
| Jenny Wren | 36 | 381 | Checkmate | — | 76 |
| Tinie | — | 55 | Silver King | — | 163 |
| Agamemnon | 36 | 380 | S. Anthony | — | 76 |
| Patience | 36 | 380 | Quackah | — | 75 |
| Ecila | 36 | 381 | Reynard | 35 | 346 |
| Lightowlers | 35 | 359 | Sally | — | 27 |
| Rouge | — | 89 | Success | — | 61 |
| Wyamaris | 37 | 366 | Caledonia | — | 52 |
| Qu'appelle | 36 | 355 | Nurse Emma | 26 | 305 |
| Nosam | 34 | 353 | Hazel | — | 20 |
| Nurse Hilda | — | 44 | Pallas | — | 48 |
| Lady Betty | 36 | 360 | Puss | — | 15 |
| Grenelle | — | 43 | Shakespear | 37 | 287 |
| Daisy | 33 | 324 | Melita | 34 | 341 |
| H. A. S. | — | 157 | Nora | — | 10 |
| A. B. C. | — | 66 | Elsie | — | 35 |
| Liz | 32 | 267 | Esperance | — 27 | 27 |

## Figures and Tables

**Fig. 1. f1:**
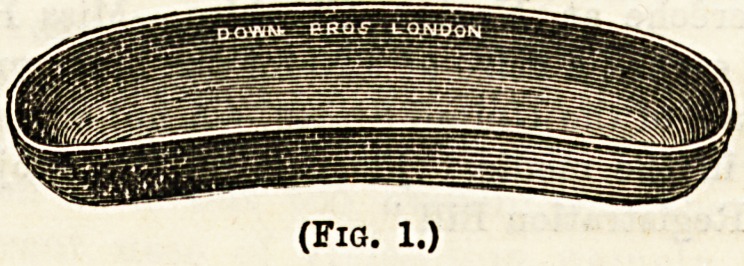


**Fig. 2. f2:**
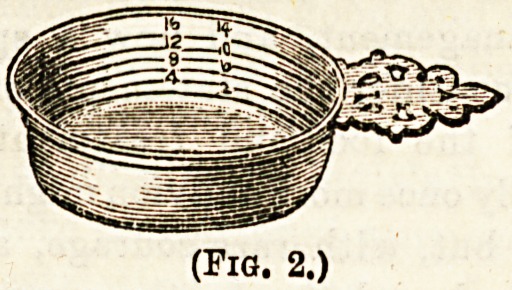


**Figure f3:**
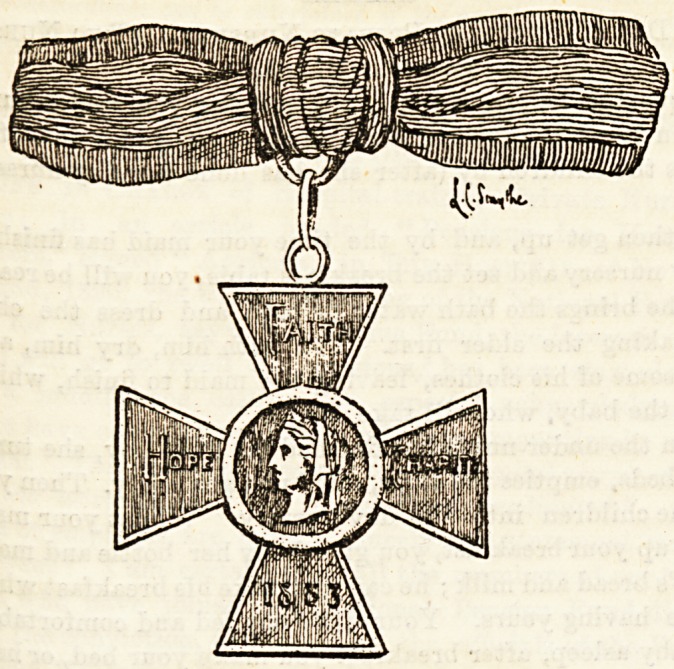


**Figure f4:**